# *Mycoplasma leachii* strain PG50 shows reduced adherence and invasion compared to *M. bovis* strains 428E and PG45

**DOI:** 10.1007/s11259-025-10901-x

**Published:** 2025-09-30

**Authors:** Daniel W. Nielsen, Samantha J. Hau, David J. Holthausen, Rohana P. Dassanayake, Bryan S. Kaplan

**Affiliations:** 1 Ruminant Diseases and Immunology Research Unit, USDA-ARS National Animal Disease Center, Ames, IA USA; 2 Virus and Prion Research Unit, USDA-ARS National Animal Disease Center, Ames, IA USA

**Keywords:** Mycoplasma leachii, Adherence, Cell culture, BVDV, Mycoplasma bovis

## Abstract

*Mycoplasma leachii* is a member of the “*Mycoplasma mycoides* cluster”, which includes the causative agent of contagious bovine pleuropneumonia (*Mycoplasma mycoides* subsp. *mycoides*). *Mycoplasma leachii* has been associated with severe outbreaks of polyarthritis, mastitis, and abortion in dairy cattle in Australia, Argentina, and China. As *M. leachii* has veterinary implications and is related to other foreign animal diseases of economic importance, we sought to investigate differences in adherence to and invasion of different bovine cells between *M. leachii* type strain PG50 and *M. bovis* strains (type strain PG45 and field strain 428E). Specifically, we tested the strains with Madin-Darby bovine kidney (MDBK), bovine turbinate (BTu), and the carticular progenitor 5 (CP5) cells for 2 and 24 h. Additionally, we tested the MDBK and BTu cells with both non-cytopathic bovine viral diarrhea virus (BVDV-1b) positive and BVDV negative fetal bovine serum. *Mycoplasma leachii* strain PG50 adhered and invaded less efficiently than *M. bovis* strains 428E and PG45 with a log_10_ reduction in adherence and invasion ranging from 1.5 to 2.9 CFU/mL. The reduced adherence and invasion of *M. leachii* type strain PG50 was observed regardless of the incubation time (2 h vs. 24 h), tested cells (MDBK, BTu, or CP5), cell type (epithelial or fibroblast), or presence of BVDV. Future work should further characterize *M. leachii* to further assess its risk to animal health and compare it phenotypically to other members of the *M. mycoides* cluster.

**Supplementary Information:**

The online version contains supplementary material available at 10.1007/s11259-025-10901-x.

## Introduction

*Mycoplasma leachii* is associated with severe outbreaks of mastitis and abortion in dairy cattle and polyarthritis in calves (Alexander et al. 1985; Chang et al. 2011; Hum et al. 2000). *M. leachii* has been detected in Australia, Argentina, and China, but there have been no reported cases in the United States (Chang et al. 2011; Hum et al. 2000; Neder et al. 2019, 2022); a thorough understanding of both the epidemiology and prevalence of *M. leachii* remains undetermined. *Mycoplasma leachii* is a member of the “*Mycoplasma mycoides* cluster”, which includes both *Mycoplasma mycoides* subsp. *mycoides* (the causative agent of contagious bovine pleuropneumonia) and *Mycoplasma capricolum* subsp. *capripneumoniae* (the causative agent of contagious caprine pleuropneumonia), which are closely related foreign animal pathogens (Manso-Silván et al. 2007; Manso-Silvan et al. 2009; Morse and Weirich 2011). Prior to the current nomenclature, *M. leachii* isolates were identified as *Mycoplasma* sp. bovine group 7 of Leach (Manso-Silvan et al. 2009). In contrast to other *Mycoplasma* spp., including other members of the mycoides cluster, *M. leachii* remains relatively understudied.

Like *M. leachii*, *Mycoplasma* (*Mycoplasmopsis*) *bovis* is also associated with polyarthritis, mastitis, and abortion; however, unlike *M. leachii*, *M. bovis* is endemic in the United States and is an important pathogen of the bovine respiratory disease complex, which is estimated to cost the U.S. cattle industry $800–900 million annually (Gaudino et al. 2022; Kamel et al. 2024). Previous work found that *M. bovis* can both adhere to and invade the Madin-Darby bovine kidney (MDBK) cell line and bovine embryonic turbinate cells (BTu) cells (Josi et al. 2018; Yacoub et al. 2025). Prior work also examined the adhesion and invasion ability of *M. bovis* in MDBK epithelial cells using a multiplicity of infection (MOI) of 10 and 50 and found that *M. bovis* strains obtained from diseased and asymptomatic cattle and bison were less capable of invasion and replication compared to *Mycoplasma bovirhinis* and *Mycoplasma bovigenitalium* strains (Yacoub et al. 2025). *Mycoplasma bovis* attaches to embryonic bovine lung cells (EBL) in cell culture (Sachse et al. 1993) and attachment reaches saturation at an MOI of 225 (Sachse et al. 1996).

Considering both the clinical implications and relatedness to other foreign diseases of economic importance and biosecurity concerns, this study investigates differences in adherence and invasion to different bovine cells between *M. bovis* strains (PG45 and 428E) and *M. leachii* strain PG50. Specifically, the strains were tested with MDBK and BTu epithelial cells and the carticular progenitor 5 (CP5) cells, which are articular cartilage progenitor cells with a fibroblast morphology from a Holstein-Friesian. For each cell type, *M. leachii* strain PG50 was less successful at adhering and invading compared to the *M. bovis* strains.

## Materials and methods

### Adherence and invasion assay

The MDBK and BTu cells verified to be negative for bovine viral diarrhea virus (BVDV) were obtained from the Cytology Laboratory of USDA’s National Veterinary Services Laboratories (Ames, IA). The CP5 cells were obtained from Millipore Sigma (St. Louis, MO). The three cell types were cultured in Minimum Essential Medium Eagle media (Sigma Aldrich, St. Louis, MO) supplemented with heat-inactivated fetal bovine serum (FBS), sodium pyruvate solution (Sigma Aldrich), and MEM non-essential amino acid solution (Sigma Aldrich). The cells were seeded in 12-well tissue culture treated plates (Falcon, Corning, NY). When the confluency of the cells was at least 90%, the cells were inoculated at a MOI of 10 to 150 with *M. bovis* strains PG45 or 428E or *M. leachii* strain PG50 and incubated at 37 °C with 5% CO_2_ for 2 and 24 h. Strains PG45 and PG50 (DSM 21131) were acquired from ATCC (Manassas, Virginia) and DSMZ GmbH (Braunschweig, Germany), respectively. Table 1 contains additional strain information. After incubating for 2 and 24 h in Minimum Essential Medium Eagle media supplemented with FBS, sodium pyruvate solution, and MEM non-essential amino acid solution, the wells were washed five times with phosphate-buffered saline (PBS). The PBS was subsequently removed, and 100 µL of trypsin EDTA (obtained from National Centers for Animal Health media preparation) was added to each well. Subsequently, the 12-well plate was incubated for 10 min, and 900 µL of PBS was added to each well, bringing the total volume to 1 mL per well. The solution was homogenized by pipetting, and serial dilutions were performed. The ability of the *M. bovis* and *M. leachii* strains to adhere to and invade was assessed by quantitating the colony forming units (CFU)/mL of each strain on Difco PPLO agar (Becton, Dickinson and Company) supplemented with D-glucose (Sigma Aldrich), sodium pyruvate (Sigma Aldrich), horse serum (Gibco, Grand Island, NY), ampicillin (Sigma Aldrich), and yeast extract (Fisher Scientific, Pittsburgh, Pennsylvania) by spotting 5 µL in triplicate. The plates were incubated at 37 °C with 5% CO_2_ for 48–60 h before colonies were counted.

### BVDV screening

To assess the presence of BVDV, FBS or cellular supernatant from CP5 cells was inoculated onto MDBK and BTu cells for one hour at 37 °C and 5% CO_2_ (Gibco MEM 11095 supplemented with 10% filtered and heat-inactivated FBS). Inoculum was removed, and BVDV-free complete MEM media was added. Cells were incubated for four days at 37 °C and 5% CO_2_. The cells were subsequently frozen at −80°C before they were thawed for a second passage. The cell suspension was removed and inoculated onto MDBK or BTu cells for 1 hour at 37 °C and 5% CO_2_. Inocula were removed, and complete MEM was added. Inoculated cells were incubated at 37 °C and 5% CO_2_ for 4 days before freezing at −80°C. Second passage frozen plates were subsequently thawed, and viral RNA was extracted from the cell suspension using a QIAcube with the QIAamp Viral RNA Mini QIAcube Kit (Qiagen, Hilden, Germany) following the manufacturer’s instructions. BVDV Viral detection was confirmed using the VetMAX-Gold BVDV PI Detection Kit (Applied Biosystems, Foster City, CA) using the PI28 known BVDV strain for the standard curve (Silveira et al. 2019). The genotype was determined by 5’ UTR sequencing using RT-PCR with HCV90-368 primers (Ridpath and Bolin 1998) and subsequent Sanger sequencing at the Iowa State University DNA Facility (Ames, IA). The BVDV-positive FBS and CP5 cells were identified as containing BVDV-1b.

### Bacterial culture and growth assessment

Using freezer aliquots of PG50, 428E, and PG45, serial dilutions were performed in duplicate using Difco PPLO broth supplemented with D-glucose (Sigma Aldrich), sodium pyruvate (Sigma Aldrich), horse serum (Gibco), ampicillin (Sigma Aldrich), and yeast extract (Fisher Scientific) to reach the approximate desired concentration of 1,000 CFU/mL in 3 mL. At 0, 2, 6, 24, 28, and 52 h, serial dilutions were performed with Dulbecco’s PBS (Gibco), and 5 uL was spotted on supplemented PPLO agar in triplicate. The plates were incubated at 37 °C with 5% CO_2_ for 48–60 h before colonies were counted. The process was repeated, and the log_10_ transformed counts were plotted using GraphPad Prism v. 10.4.1 (GraphPad Software, Boston, MA).

#### Statistical analysis

Six replicates were performed for the cells using BVDV-positive media. Two replicates were performed for the cells using BVDV-free media. Statistical tests and graphing were performed with GraphPad Prism v. 10.4.1. An ordinary one-way ANOVA and Tukey’s multiple comparisons were performed on the log_10_ transformed counts for both BVDV-positive (Gibco Fetal Bovine Serum Premium Plus) and BVDV-free FBS (PAA Laboratories, Toronto, Canada). The dataset is available for download (Online Resource 1). An ordinary two-way ANOVA and Tukey’s multiple comparisons were performed on the log_10_ transformed counts of the entire dataset to assess any influence of the BVDV-positive FBS. For the bacterial growth curves, an ordinary two-way ANOVA and Tukey’s multiple comparisons were performed on the log_10_ bacterial transformed counts.

**Table 1:** Metadata for *M. leachii* (PG50) and *M. bovis* strains (PG45 and 428E)

Strain	Clinical disease	Strain Year & Location	Reference(s)
PG50 (DSM 21131, NCTC 10133)	Arthritis	1963 Australia	(Simmons and Johnston 1963)
PG45 (Donetta)	Mastitis	1961 Connecticut, USA	(Askaa & Erno, 1976; Hale et al. 1962)
428E	Arthritis, pneumonia	1996 Iowa, USA	(Adegboye et al. 1996)

Online Resource 1 A spreadsheet containing the log_10_ transformed counts for both BVDV-positive and BVDV-free FBS datasets.

## Results

### Adherence to and invasion of MDBK, BTu, and CP5 cells

**Fig. 1:**
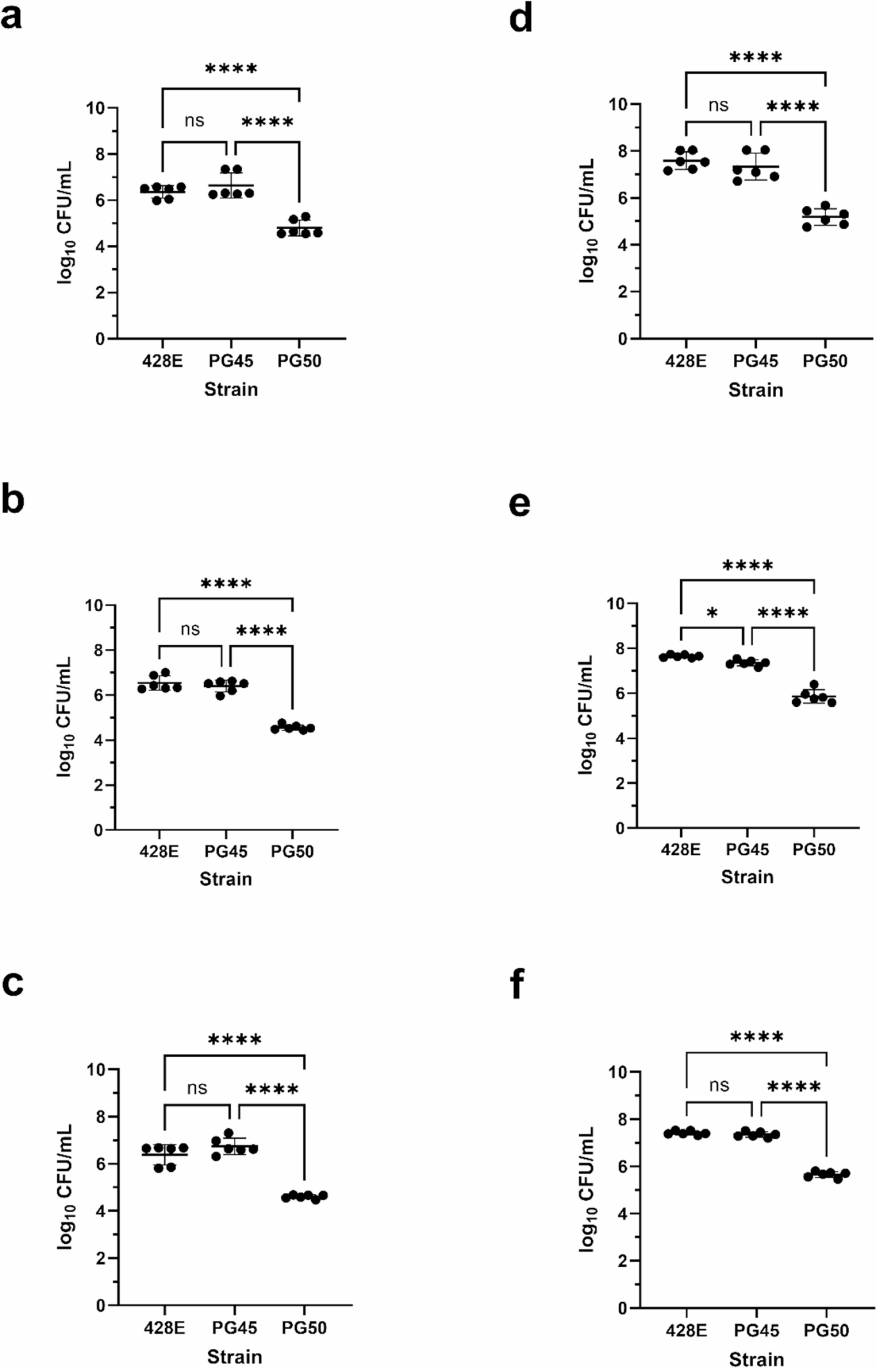
Adherence and invasion of *Mycoplasma* species in BVDV-positive cells. At two hours, *M. leachii* strain PG50 adhered and invaded less successfully than *M. bovis* strains 428E and PG45 to MDBK (**a**), BTu (**b**), and CP5 (**c**) cells in BVDV-positive media (*p* < 0.0001). At 24 h, *M. leachii* strain PG50 also adhered and invaded less successfully than *M. bovis* strains 428E and PG45 to MDBK (**d**), BTu (**e**), and CP5 (**f**) cells in BVDV-positive media (*p* < 0.0001). Error bars indicate the mean with standard deviation. Statistical significance is denoted as *P* < 0.0001: ****; *P* < 0.001: ***; *P* < 0.01:**; *P* < 0.05:*

At two hours, *M. leachii* strain PG50 was less likely to adhere to and invade the MDBK and BTu epithelial cells compared to *M. bovis* strains 428E (*p* < 0.0001) and PG45 (*p* < 0.0001) (Fig. 1a and b, respectively). This difference was also observed for the CP5 cells, a cartilage progenitor (*p* < 0.0001), suggesting that the difference in adherence is not epithelial cell specific (Fig. 1c). Similarly, at 24 h, *M. leachii* strain PG50 was less likely to adhere to and invade MDBK, BTu, and CP5 cells compared to the *M. bovis* strains (*p* < 0.0001) (Fig. 1d–f, respectively). Regardless of timepoint, *M. leachii* strain PG50 demonstrated reduced adherence and invasion compared to *M. bovis* strains PG45 and 428E.

When comparing the adherence to and invasion of each mycoplasma among the tested cells, each mycoplasma, with respect to itself, adhered to and invaded MDBK, BTu, and CP5 cells equivalently at 2 h (Fig. S1a, S1b, and S1c). At 24 h, *M. bovis* strains adhered to and invaded equivalently to MDBK, BTu, and CP5 cells (Fig. S1d and S1e), but *M. leachii* strain PG50 adhered and invaded significantly better to CP5 (*p* < 0.05) and BTu (*p* < 0.01) cells compared to MDBK cells (Fig. S1f).

**Fig. 2:**
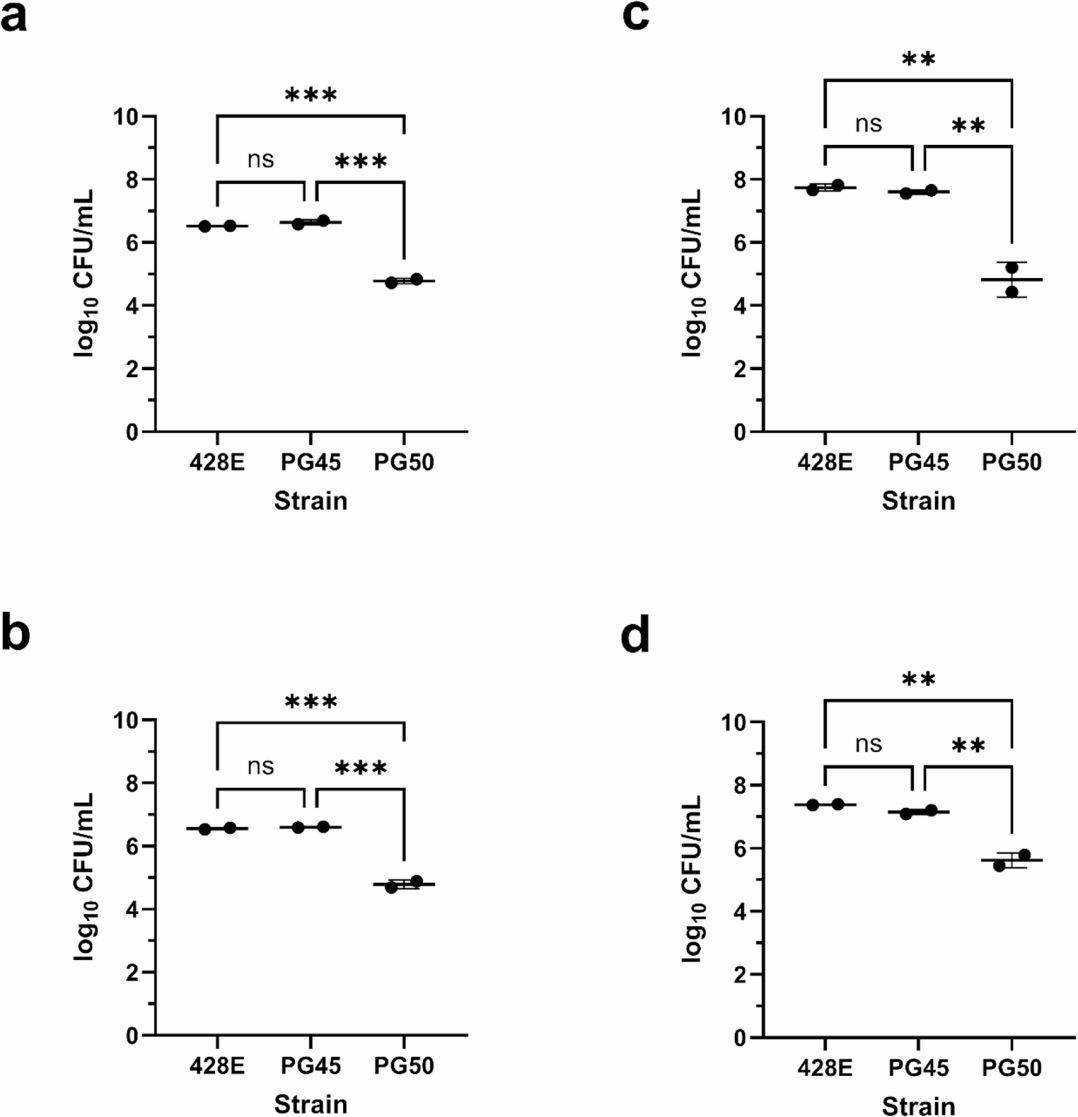
Adherence and invasion of *Mycoplasma* species in BVDV-free cells. At two hours, *M. leachii* strain PG50 adhered and invaded less successfully than *M. bovis* strains 428E and PG45 to MDBK (**a**) and BTu (**b**) cells in BVDV-free media (*p* < 0.001). At 24 h, M. *leachii* strain PG50 also adhered and invaded less successfully than *M. bovis* strains 428E and PG45 to MDBK (**c**) and BTu (**d**) cells in BVDV-free media (*p* < 0.01). Error bars indicate the mean with standard deviation. Statistical significance is denoted as *P* < 0.0001: ****; *P* < 0.001: ***; *P* < 0.01:**; *P* < 0.05:*

As BVDV is also commonly detected in laboratory grade FBS used in commercial vaccines and research (Gomez-Romero et al. 2021; Pastoret 2010; Xia et al. 2011), screening of BVDV was performed. Screening identified that initial experiments were done using serum containing a non-cytopathic BVDV. Sequence analysis of the 5` untranslated region revealed the BVDV subtype 1b (BVDV-1b). Additionally, the CP5 cells were found to be infected with non-cytopathic BVDV-1b. Given literature suggesting BVDV results in host cell differences compared to BVDV-negative cell lines, including cellular transcriptional changes (Liu et al. 2019; Neill and Ridpath 2003; Villalba et al. 2016), we assessed the adherence to and invasion of MDBK and BTu cells to *M. leachii* and *M. bovis* strains using BVDV-free FBS. Similar to the BVDV-positive media, at two hours, *M. leachii* strain PG50 was less likely to adhere to and invade MDBK (Fig. 2a) and BTu (Fig. 2b) cells compared to *M. bovis* strains 428E and PG45 (*p* < 0.001). At 24 h, *M. leachii* strain PG50 was also less likely to adhere to and invade MDBK (Fig. 2c) and BTu (Fig. 2d) cells compared to the *M. bovis* strains (*p* < 0.01). Like the BVDV-positive results, the BVDV-free results demonstrate that *M. leachii* strain PG50 adheres and invades less successfully than *M. bovis* strains PG45 and 428E. There were no statistical differences between the BVDV-positive and BVDV-free results, suggesting that BVDV did not influence the adherence and invasion (Fig. S2).

## Growth assessment

**Fig. 3:**
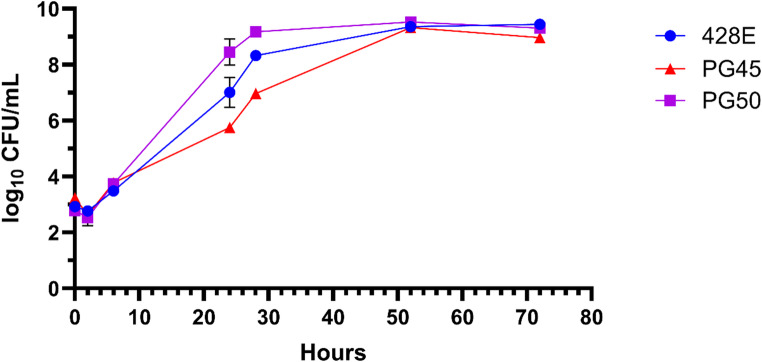
Growth curve of *M. bovis* (strains 428E and PG45) and *M. leachii* (PG50) in supplemented PPLO media. The strains are represented by blue circles (428E), red triangles (PG45), and purple squares (PG50). Error bars indicate the standard error of the mean. *Mycoplasma leachii* PG50 grew faster than *M. bovis*, resulting in 1.44 and 2.7 log_10_ CFU/mL greater than *M. bovis* strains 428E and PG45 at 24 h, respectively

Growth rate analysis of the mycoplasmas revealed that *M. leachii* had a faster growth rate than the *M. bovis* strains resulting in increased counts at 24 (*p* < 0.05) and 28 (*p* < 0.001) hours. By 24 h of culture, *M. leachii* had reached 8.4 × 10^8^ CFU/mL in supplemented PPLO broth (Fig.3). Between 24 and 28 h, *M. leachii* surpassed 1 × 10^9^ CFU/mL; in contrast, the *M. bovis* strains did not reach 1 × 10^9^ CFU/mL until 52 h of culture (Fig. 3).

## Discussion

The literature has established the ability of *M. bovis* to adhere to and invade different cell lines (Burgi et al. 2018; Josi et al. 2018; Sachse et al. 1993, 1996; Thomas et al. 2003; van der Merwe et al. 2010; Yacoub et al. 2025), and prior work has also shown the relative difference of other mycoplasmas to adhere and/or invade compared to *M. bovis*. For example, previous work found *M. bovis* strains obtained from both diseased and asymptomatic cattle and bison were less capable of invasion and replication in MDBK cells compared to *M. bovirhinis* and *M. bovigenitalium* strains (Yacoub et al. 2025). Previous work has also demonstrated that *M. mycoides* subsp. *mycoides* is less effective at invading and surviving inside bovine macrophages compared to *M. bovis* (Totté et al. 2024). Notably, *M. mycoides* subsp. *mycoides* has also been shown to exhibit little strain variation in adherence capability to cattle lung epithelial cells (Aye et al. 2015).

Attachment and invasion are crucial steps in the establishment of disease by many pathogens, including mycoplasmas (Xiu et al. 2024). Here, we assessed the ability of *M. leachii* strain PG50 to adhere to and invade MDBK, BTu, and CP5 cells relative to *M. bovis*, as *M. leachii* is a relatively understudied pathogen relevant to animal health. We also assessed if the presence of BVDV in the cell culture media influenced adherence and invasion by *M. bovis* or *M. leachii*.

In this study, *M. leachii* strain PG50 adhered and invaded less successfully than the *M. bovis* strains. This result appeared to be independent of the growth dynamics of the strains, as *M. leachii* strain PG50 had a faster growth rate compared to the *M. bovis* strains. The reduced adherence and invasion by PG50 could suggest that *M. leachii* may be less pathogenic compared to *M. bovis*; however, prior work investigating *M. bovis* strains obtained from diseased and asymptomatic cattle and bison indicated no correlation between adherence and clinical disease association (Yacoub et al. 2025). In this work, we were unable to differentiate adherence from the combined adherence and invasion data of the mycoplasmas. However, previous work has demonstrated that *M. mycoides* subsp. *mycoides* is less effective at invading and surviving inside bovine macrophages compared to *M. bovis* (Totté et al. 2024). Considering this work, it suggests that the reduced adherence and invasion result by *M. leachii* could be due to a reduction of invasion. However, it is worth noting that this study used epithelial and fibroblast cells rather than monocyte derived macrophages. Future work should investigate any similarities in invasion capabilities between *M. leachii and M. mycoides* subsp. *mycoides*.

Here, we found that the pattern among the *M. bovis* strains and *M. leachii* strain PG50 was consistent across the tested cells with greater counts of *M. bovis* recovered for MDBK, BTu, and CP5 cells. Prior work has found that *M. mycoides* subsp. *mycoides* was able to bind with great efficiency to lung epithelial cells and poorly to fibroblasts (Aye et al. 2015). We found that the *M. leachii* and *M. bovis* strains adhered to and invaded similarly with respect to each strain among the MDBK, BTu, and CP5 cells at 2 h. At 24 h, the *M. bovis* strains continued this trend, but *M. leachii* strain PG50 had greater adherence and invasion to CP5 (fibroblast) and BTu (epithelial) cells compared to MDBK cells (epithelial). Together, this suggests that binding to the different cells by *M. leachii* may be time dependent or specific to certain cells, instead of being a specific cell type (epithelial vs. fibroblast). However, the MDBK, BTu, and CP5 cells were not run concurrently, and we did not investigate adult bovine lung cells, which were used in the previous work (Aye et al. 2015). Further work is needed to determine if the adherence pattern of *M. leachii* is representative of other *Mycoplasma mycoides* cluster agents and whether *M. leachii* adheres and invades more efficiently to other cell types, such as macrophages and bovine lung cells.

Testing of the bovine serum and CP5 cells indicated they were positive for non-cytopathic BVDV-1b. To assess the impact of BVDV exposure on the adherence and invasion of *M. leachii* and *M. bovis*, we evaluated adherence and invasion in the MDBK and BTu cells using BVDV-free media. The results indicated that there were no statistically significant differences between the MDBK and BTu cells between the BVDV-positive and BVDV-free conditions. This corresponds with prior work by Burgi et al., in which BVDV-positive and BVDV-free bovine macrophages (BoMac) cells had no differences in the levels of uptake and growth of *M. bovis* (Burgi et al. 2018). Although BVDV is known to alter gene expression in some cell lines (Liu et al. 2019; Neill and Ridpath 2003; Villalba et al. 2016), our work showed no difference in adherence to and invasion of BVDV exposed and unexposed cells. However, the BVDV strain identified in this study was a non-cytopathic subtype 1b strain, and it is unclear if a different subtype or a cytopathic strain of BVDV would alter the outcome of this study.

In conclusion, *M. leachii* strain PG50 demonstrated consistently lower adherence and invasion capabilities compared to *M. bovis* strains 428E and PG45. This consistently lower adherence and invasion occurred regardless of the incubation time (2 h vs. 24 h), different tested cells (MDBK, BTu, or CP5), cell type (epithelial or fibroblast), or presence of BVDV-1b. Future work should further characterize *M. leachii* to assess its risk to animal health and any phenotypic similarities to other members of the *M. mycoides* cluster.

## Supplementary Information

Below is the link to the electronic supplementary material.

Supplementary Material 1 (XLSX 14.3 KB)

Figure S1 Two-way ANOVA of bacterial strains in BVDV-positive media and BVDV-negative media with MDBK cells at 2 h (a) and 24 h (b) and BTu cells at 2 h (c) and 24 h (d). Error bars indicate the mean with standard deviation. (PNG 104 KB)

High Resolution Image (TIF 1.12 MB)

Figure S2 One-way ANOVA of bacterial strains 428E (a), PG45 (b), and PG50 (c) at 2 h and 428E (d), PG45 (e), and PG50 (f) at 24 h in BVDV-positive media. The only statistical difference observed was for PG50 at 24 h (f). Error bars indicate the mean with standard deviation. Statistical significance is denoted as *P* < 0.01:**; *P* < 0.05:*. (PNG 177 KB)

High Resolution Image (TIF 3.48 MB)

## Data Availability

Data is provided within the manuscript or supplementary information files.
