# Supplementary figures and images for: Mycoplasma leachii strain PG50 shows reduced adherence and invasion compared to M. bovis strains 428E and PG45
Source: Vet Res Commun. 2025 Sep 30;49(6):339. doi: 10.1007/s11259-025-10901-x (PMC12484092; doi:10.1007/s11259-025-10901-x)

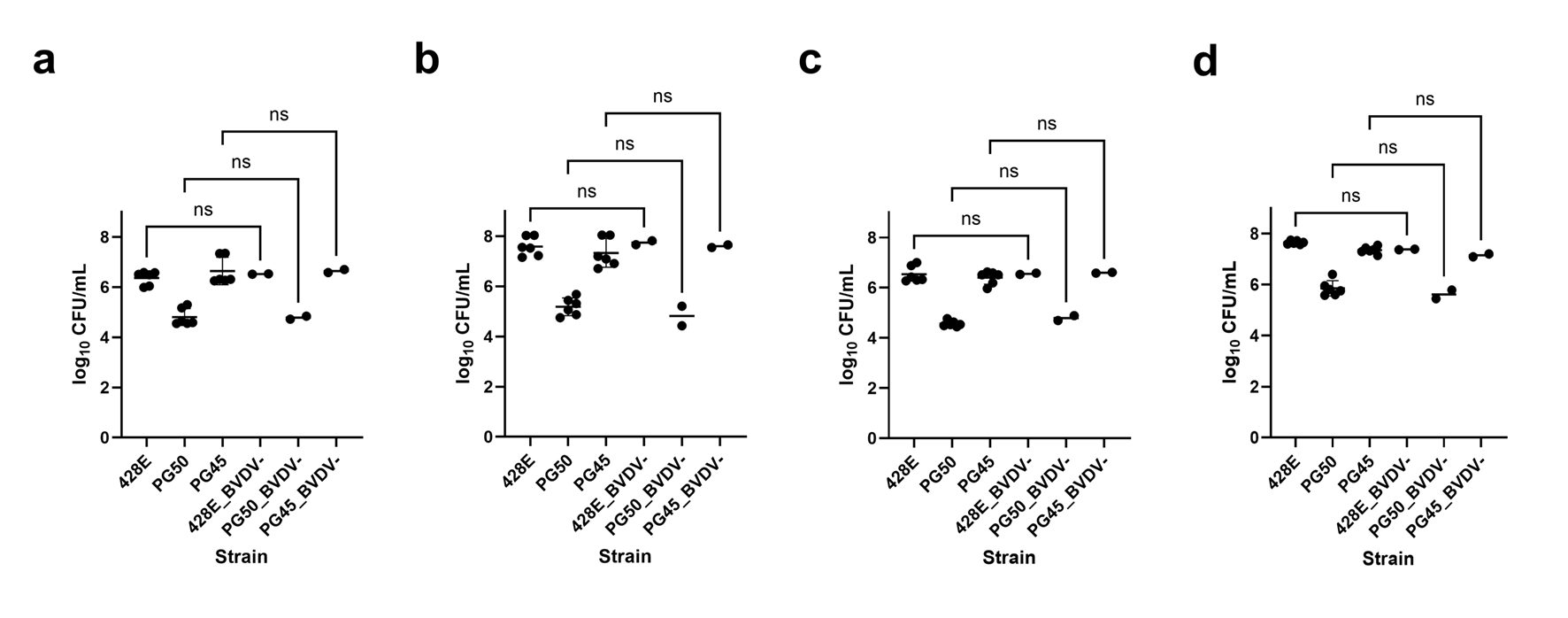

Supplement: Supplementary file 2 — Two-way ANOVA of bacterial strains in BVDV-positive media and BVDV-negative media with MDBK cells at 2 h (a) and 24 h (b) and BTu cells at 2 h (c) and 24 h (d). Error bars indicate the mean with standard deviation. (PNG 104 KB) [file 11259_2025_10901_Fig4_ESM.png]

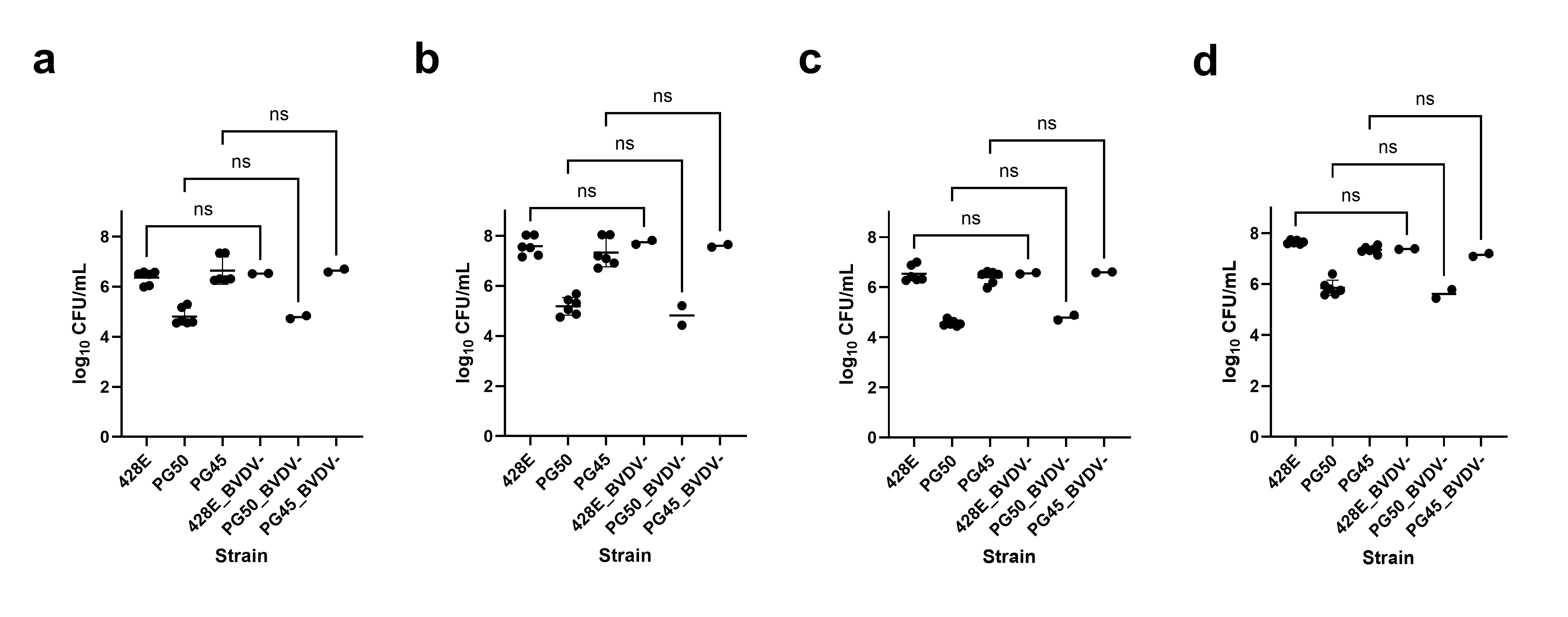

Supplement: Supplementary file 3 — High Resolution Image (TIF 1.12 MB) [file 11259_2025_10901_MOESM2_ESM.tif]

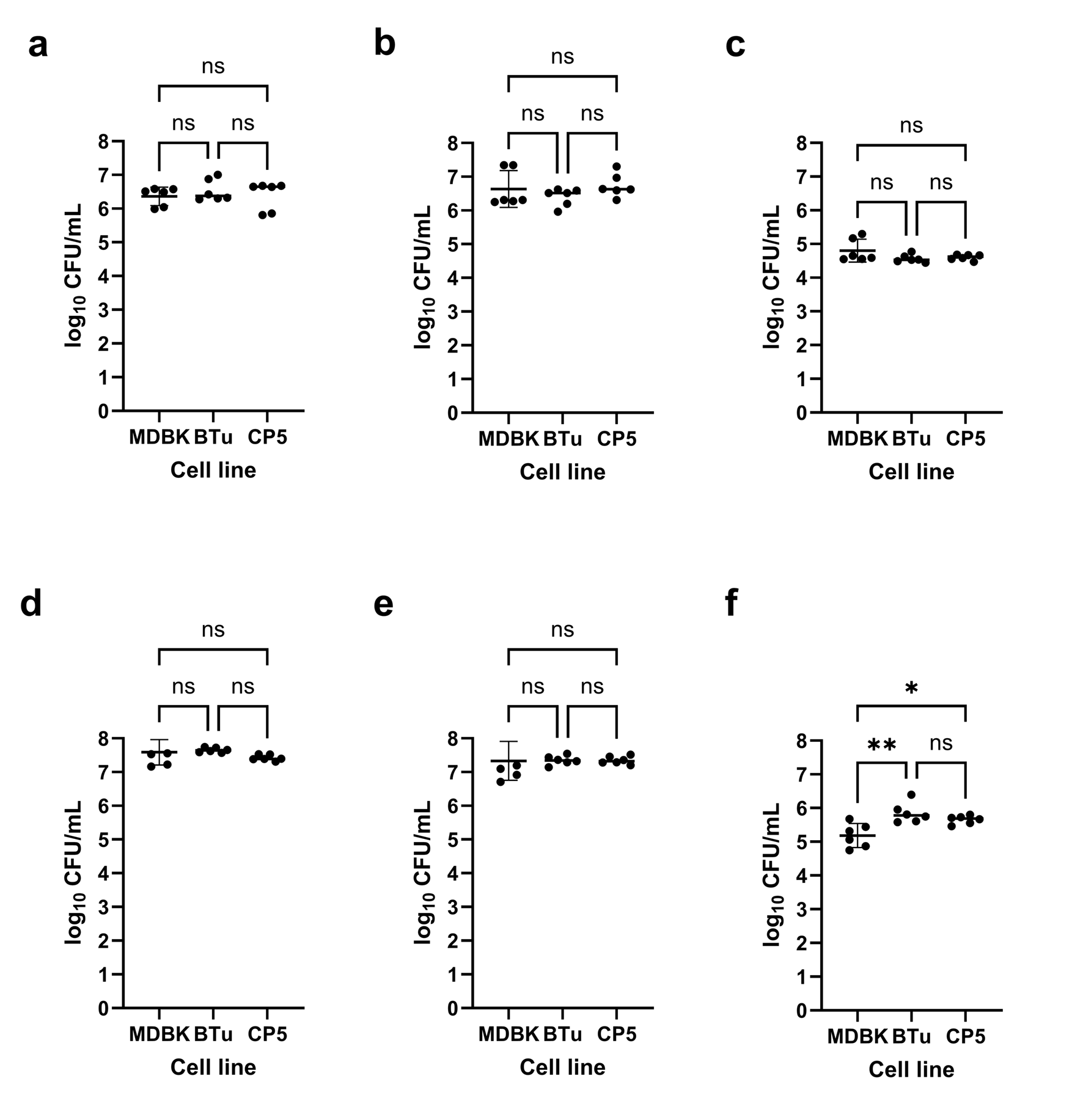

Supplement: Supplementary file 4 — One-way ANOVA of bacterial strains 428E (a), PG45 (b), and PG50 (c) at 2 h and 428E (d), PG45 (e), and PG50 (f) at 24 h in BVDV-positive media. The only statistical difference observed was for PG50 at 24 h (f). Error bars indicate the mean with standard deviation. Statistical significance is denoted as P < 0.01:**; P < 0.05:*. (PNG 177 KB) [file 11259_2025_10901_Fig5_ESM.png]

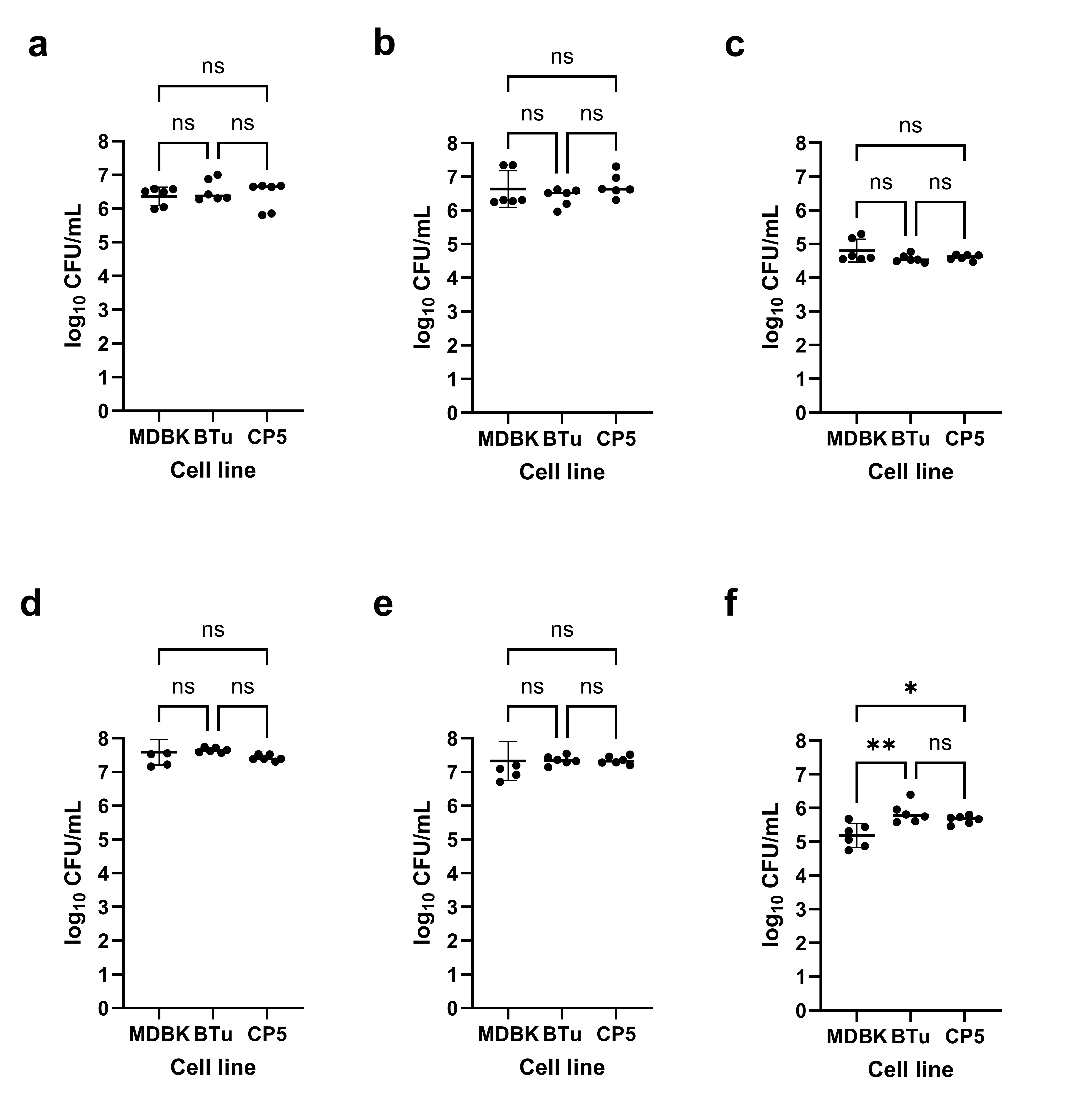

Supplement: Supplementary file 5 — High Resolution Image (TIF 3.48 MB) [file 11259_2025_10901_MOESM3_ESM.tif]
